# G3 2025 Reviewer Index

**DOI:** 10.1093/g3journal/jkaf258

**Published:** 2025-12-10

Below is a list of individuals who have reviewed manuscripts for G3: Genes|Genomes|Genetics during the preparation of Volume 15 (2025). The aim of G3: Genes|Genomes|Genetics is to communicate high-quality, well-described, useful research, across all areas of genetics and genomics. To succeed, we must recognize what meets our criteria for publication, making sure the presentation is accurate, unambiguous, and easily readable. For this, we depend heavily upon our reviewers, who perform this indispensable service without reward. The quality of the journal strongly reflects the quality of their efforts. We apologize to any reviewer whose name has been inadvertently omitted, and we are sincerely grateful to all.

Abbasi, Burhan Ud Din

Aberlenc-Bertossi, Frédérique

Abitua, Phil

Ables, Elizabeth T.

Ablondi, Michaela

Abueg, Linelle

Abusleme, Loreto

Adams, Richard

Adamson, Kalev

Adelman, Zach N.

Adhikari, Prakriti

Aguillon, Stepfanie

Ahlawat, Neetika

Ahmad, Kami

Ahmed, Muhammad

Ahmed, Shawn

Ahmed-Braimah, Yasir H.

Akakpo, Roland

Akbari, Omar S.

Akdemir, Deniz

Akopyan, Maria

Al-Jumaili, Ahmed S.

Alahmad, Samir

Albert, Frank W.

Albert, Nick

Albert, Victor

Albrechtsen, Anders

Aldiss, Zachary

Alemu, Admas

Alfenas, Acelino Couto

Alfoldi, Jessica

Ali, Ahmad

Alioto, Tyler S.

Allaband, Celeste

Alladassi, Boris M.E.

Allal, François

Allwood, Julia

Almeida, Alexandre

Alonso-Garcia, Marta

Alphey, Luke

Alves Aflitos, Saulo

Amadeu, Rodrigo R.

Amundson, Kirk

Anche, Mahlet T.

Andersen, Erik C.

Anderson, Jill T.

Andersson, Martin N.

Andre, Catherine

Andreassen, Ole A.

Andres, Aida

Andrew, Rose L.

Anstead, Claire

Anttila, Verneri

Apfeld, Javier

Appels, Rudi

Arama, Eli

Aranda, Manuel

Arbeitman, Michelle N.

Arbelaez, Juan D.

Archibald, Alan L.

Arge, Luis W.

Arguello, J. Roman

Arick, Mark A.

Arimoto, Asuka

Armstrong, Ellie

Armstrong, Ellie E.

Arndt, Karen M.

Arur, Swathi

Ashrafi, Hudson

Åstrand, Johanna

Augustinos, Antonios

Avila, Felipe

Awe, Olaitan I.

Ayroles, Julien F.

Babbitt, Patricia

Babbucci, Massimiliano

Bach, Erika A.

Badea, Tudor C.

Badet, Thomas

Baer, Charles F.

Bagley, Robin K.

Bahmdorff, Simon

Bahn, Yong-Sun

Bai, Fengyan

Bai, Xiaofei

Bai, Yuling

Bajgar, Adam

Bakkeren, Guus

Bakshi, Asif A.

Balbontin, Roberto

Balding, David

Baldridge, Dustin

Baldwin, Thomas

Baldwin-Brown, James G.

Baltrus, David A.

Bambah-Mukku, Dhananjay

Bandyadka, Shruthi

Bannasch, Danika

Baquero, Enrique

Baquero, Fernando

Baranski, Matt

Barash, Yoseph

Barbash, Daniel A.

Barcaccia, Gianni

Barchi, Lorenzo

Bardol, Nicolas

Baril, Tobias

Barkai, Naama

Barley, Anthony

Baroncelli, Riccardo

Barr, Maureen M.

Barrera-Redondo, Josué

Barrick, Jeffrey E.

Baschien, Christiane

Bassik, Michael

Bassil, Nahla V.

Bastian, Frederic Bastian

Bastos de Oliveira, Francisco M.

Batista-García, Ramon

Batley, Jacqueline

Baurain, Denis

Bautista, Oscar

Bavik, Linnea

Bayfield, Mark

Beaulieu, Jean

Becton, Hope R.

Bedinger, Patricia

Beh, Christopher

Behmer, Spence

Beier, Sebastian

Bell, Rayna

Belsare, Saurabh

Ben Tov, Daniela

Bennett, Richard J.

Benoit, Joshua B.

Benoit Gelber, Isabelle

Bensinger, Steven J.

Bentlage, Bastian

Bergey, Christina

Bergmeier, Franziska

Bernardi, Giacomo

Berro, Maria Inés

Berthouly-Salazar, Cécile

Bertolini, Edoardo

Besson, Mathieu

Betancourt, Andrea J.

Bevilacqua, Philip

Bhamidipati, Sridevi

Bhandari, Prashant

Bhar, Monmita

Bhat, Shripad R.

Bhattacharjee, Shatabdi

Bhattacharya, Aniket

Bhattacharya, Arjun

Bhattacharyya, Arindam

Bhattarai, Upendra R.

Bianco, Luca

Billings, Grant T.

Billmyre, R. B.

Birchler, James A.

Bird, Phil

Birol, Inanç

Bishop, Anusha

Biteau, Benoit

Bitter, Mark

Blackall, Linda

Blackburn, David

Blackmon, Heath

Blackshaw, Seth

Blacque, Oliver E.

Blair, Matthew W.

Bland, Michelle

Blaxter, Mark L.

Blay, Natalia

Bleidorn, Christoph

Blencowe, Ben

Blumenstiel, Justin P.

Bocak, Ladislav

Boehm, Frederick J.

Boehnke, Michael

Bogdanov, Mikhail

Boggess, Sarah L.

Bolon, Daniel N.

Bonifazi, Renzo

Bono, Hidemasa

Booth, Warren

Borde, Valerie

Borin, Marcello

Borreguero-Munoz, Nerea

Bosch, Justin A.

Bouchet, Sophie

Bouffier, Laurent

Boulin, Thomas

Boulton, Rebecca A.

Bourtzis, Kostas

Boutros, Michael

Boyer, Alexandre

Braasch, Ingo

Bracewell, Ryan R.

Brachi, Benjamin

Bradshaw, Alex J.

Braendle, Christian

Brahma, Sandipan

Brand, Philipp

Brans, Kristien

Branstetter, Michael G.

Brar, Gloria A.

Brault, Charlotte

Bravo Núñez, María Angelica

Brehm, Ali

Bretman, Amanda

Breton, Sophie

Brewer, Bonita J.

Brewer, Marin

Bringmann, Henrik

Brinkmeyer-Langford, Candice

Brito, Luiz F.

Brodsky, Michael H.

Brooks, Samantha

Brown, C. Titus

Brown, Margaret

Brown, Matthew

Brown, Thomas

Brown-Guedira, Gina

Bruce, Ashley

Brueggeman, Robert

Buchon, Nicolas

Buck, Michael J.

Buck, Ryan

Budak, Hikmet

Budeanu, Marius

Buell, C. Robin

Buerger, Reinhard

Buffalo, Vince

Bui Quang, Minh

Bullock, Simon

Burda, Joshua

Burga, Alejandro

Burke, Gaelen R.

Burke, Molly

Burridge, Paul W.

Burten, Rachel B.

Bus, Vincent G.

Bushley, Kathryn E.

Bushman, B. S.

Bustos-Korts, Daniela

Caesar, Lilian

Cagan, Alex

Cahan, Sara H.

Cahill, James

Caldwell, Guy A.

Calvi, Brian R.

Cameron, Ellen

Campa Negrillo, Ana

Campbell, Polly

Campbell, Todd

Cano, Liliana M.

Cantet, Rodolfo J.

Cantù, Dario

Cao, Wei

Capkova Frydrychova, Radmila

Caproni, Leonardo

Carbone, Mary A.

Card, Daren

Card, Gwyneth M.

Cardinale, Daniela

Cardoso Moreira, Margarida

Carleton, Bruce

Carmichael, S Thomas

Carneiro, Miguel

Carr, Erin C.

Carrasquinho, Isabel

Carthew, Richard W.

Cassone, Bryan J.

Castaño, Maria

Castoe, Todd A.

Castrignanò, Tiziana

Catchen, Julian M.

Caulfield, Jasmine

Cavalli, Gioacomo

Cazenave, Xabi

Centanin, Lazaro

Chaaban, Amanda

Chain, Frédéric J.

Chakraborty, Mahul

Chalasani, Sreekanth H.

Chamberlin, Helen M.

Chamorro, Lourdes

Chancellor, Jordan L.

Chandler, Ron

Chang, Ching-Ho

Chang, Mingchang

Chapman, Mark A.

Charcosset, Alain

Charlat, Sylvain

Charlesworth, Deborah

Chavan, Ankita

Chaves, Saulo

Chavez, Daniel

Chedin, Frederic

Chen, Julian

Chen, Mingjie

Chen, Ningbo

Chen, Qiuyue

Chen, Shanyuan

Chen, Sharon

Chen, Shihui

Chen, Zhiqiang

Chen, Zhuo

Cheng, Hao

Cheng, Xiaoheng

Cherif, Emira

Chetelat, Roger T.

Chevin, Luis-Miguel

Childers, Anna K.

Childs, Kevin L.

Childs-Disney, Jessica L.

Chin-Sang, Ian

Chiolo, Irene

Cho, Ken W.Y.

Choi, Jiyeon

Chopra, Kanwaljit

Choudhary, Mukesh

Christensen, Kris A.

Christensen, Ole F.

Christoph Liedtke, Hans

Chu, Ye

Chudalayandi, Sivanandan

Chueca, Luis J.

Chung, Henry

Chung, SeYeon

Churchill, Gary A.

Ciruna, Brian

Civetta, Alberto

Clark, Andrew G.

Clark, Rene

Clark-Cotton, Manuella

Clarkson, Chris

Claycomb, Julie M.

Clough, Steven

Coate, Jeremy

Cobo, Carmen

Coelho, Marco A.

Cohen, Stephen P.

Colaiácovo, Monica P.

Colasanti, Joseph

Colston, Timothy J.

Comai, Luca

Comeault, Aaron

Conradt, Barbara

Cook, Tiffany

Coque, Teresa M.

Corbett, Anita H.

Cordero, Julia B.

Cormack, Brendan

Cornille, Amandine

Corradi, Nicolas

Corsi, Ann K.

Cortesero, Anne Marie

Cortinovis, Gaia

Cosacak, Mehmet I.

Coskun, Peren

Cosseau, Céline

Cournac, Axel

Coutinho-Budd, Jaeda

Covarrubias-Pazan, Giovanny

Cowen, Leah E.

Cox, Brandon

Cox, Nancy J.

Craig, Ann Marie

Cramer, Robert A.

Crawford, Andrew J.

Crawford, Lorin

Cresko, William A.

Cristancho, Marco A.

Croft, Amanda

Croll, Daniel

Crossa, José

Crouch, Jo A.

Crowley, Liam

Cruppe, Giovana

Cuello, Clément

Cummins, Scott F.

Cunningham, Kyle W.

Cuomo, Christina A.

Curry, Caitlin

Cussiol, José Renato R.

Cutter, Asher D.

D'Amico-Willman, Katherine M.

Dabi, Amjad

Dagar, Sumit Singh

Dahanukar, Anupama A.

Daida, Kensuke

Dainat, Benjamin

Dainat, Jacques

Dal'Sasso, Thais

Dallaire, Alexandra

Dallas, Jason

Dallman, Julia

Damschroder, Deena

Darrington, Michael

Das, Debabrata

Davenport, Emily

David-Palma, Marcia

Davis, Brian W.

Davis, Richard

Davison, Angus

Dawe, R. K.

Dawson, Harry

De, Titir

De Fine Licht, Henrik H.

De Koning, A.P. Jason

De Koning, Dirk-Jan

De la Mata, Raul

De Leon, Natalia

De Medeiros, Bruno

De Vega, Jose

De Verdal, Hugues

De Vos, Lieschen

De Vries, Ronald P.

De Wit, Pierre

Deal, Roger B.

Dearing, Denise

Debes, Paul V.

Decker, Jared E.

Degen, Bernd

DeGiorgio, Michael

Degnan, Patrick

Degregori, Sam

Dekker, Job

Dekker, Teun

Del Bem, Luiz-Eduardo

DeLuna, Alexander

Demissie, Zerihun

Demuth, Jeff

Den Hollander, Anneke I.

Deng, Yangqing

Deng, Yun

Deng, Zhanao

Deraje, Puneeth

Derry, W. B.

Derry, W. Brent W.

Des Marais, David

Destanović, Dalila

Dettman, Jeremy R.

Dewan, Adam

Dewey, Colin

Dhillon, Braham

Dhungana, Singha

Di Antonio, Marco

Di Martino, Patrick

Dias, Guilherme B.

Dias, Kaio O.

Dias, Luiz A.

Diaz, Patricia

Diaz, Raul

Díaz Escandón, David

DiCenzo, George C.

Dickinson, Amanda

Dickinson, Daniel J.

Diepenbrock, Christine H.

Diez, Michay

Difford, Gareth F.

Diggle, Stephen

Dilkes, Brian P.

Dillon, Marcus M.

Dineen, Lauren

Dinesh-Kumar, Savithramma

Ding, Yiliang

Ding, Zhihao

Diouf, Esther

Disney, Matthew D.

Dittmann, Elke

Dittrich, Carolin

Doench, John

Doering, Tamara L.

Doligez, Agnès

Dominguez, Daniel

Donczew, Rafal

Dong, Emily

Dong, Ke

Dong, Qiang

Dorus, Steve

Doucet O'Hare, Tara T.

Douglas, Peter

Dreisigacker, Susanne

Driscoll, Monica

Drögemüller, Michaela

Druet, Tom

Druzhinina, Irina

Du, Li-Lin

Du, Rui

Duan, Bingbing

Duarte, Diana

Dudchenko, Olga

Dudycha, Jeff

Duenk, Pascal

Dufresne, France

Dumont, Beth L.

Dumonteil, Eric

Dunham, Maitreya J.

Dunning, Luke

Duperron, Sébastien

Duplessis, Sebastien

Durbin, Richard

Duret, Laurent

Duvall, Laura B.

Duvaux, Ludovic

Dyhrman, Sonya

Dykhuizen, Emily

Ebel, Emily R.

Econopouly, Bethany F.

Edge, Michael D.

Edger, Patrick P.

Edvardsen, Rolf

Ehrenreich, Ian M.

Eichler, Evan E.

El-Kassaby, Yousry A.

El-Kebir, Mohammed

Elaswad, Mohamed T.

Ellis, David A.

Emami-Khoyi, Arsalan

Emerson, J. J.

Emery, Gregory

Enciso-Maldonado, Guillermo Andrés

Endelman, Jeffrey B.

Enduru, Nitesh

Engebrecht, JoAnne

Engelbrecht, Juanita

Englert, Christopher

Erickson, James W.

Erogula, Cagla

Errbii, Mohammed

Escalona, José

Escalona, Merly M.

Esfandyari, Hadi

Eskin, Eleazar

Espenshade, Peter

Espregueira Themudo, Gonçalo

Esuma, Williams

Evans, Ben J.

Evans, Matthew M.

Evans, Steven N.

Everhart, Sydney E.

Ewe, Chee Kiang

Excoffier, Laurent

Extavour, Cassandra G.

Eyre-Walker, Adam

Faddeeva-Vakhrusheva, Anna

Faircloth, Brant C.

Fang, Jinggui

Fang, Lingzhao

Fang, Zhongming

Fankhauser, Christian

Farber, Charles

Farrer, Rhys A.

Fausett, Sarah

Fay, Justin C.

Feany, Mel B.

Fearnhead, Paul

Feil, Edward J.

Feldman, Jessica L.

Félix, Marie-Anne

Fellers, John P.

Feng, Chao

Feng, Siyuan

Feng, Zhiyong

Fenton, Andy

Ferguson-Smith, Anne

Ferkey, Denise M.

Fernandes, Samuel B.

Fernandez, Gina E.

Fernández-Pavía, Sylvia

Ferrão, Luis Felipe V.

Ferreiro, Alejandro M.

Fewer, David

Ficke, Andrea

Ficklin, Stephen

Figueras, Antonio

Filatov, Dmitry A.

Filiault, Daniele

Findlay, Brandon

Finkers, Richard

Fire, Andrew

Fischer, Mareike

Fisher, Kaitlin J.

Fitz-Gibbon, Sorel

Fitzpatrick, David A.

Flagel, Lex

Flatt, Thomas

Fleißner, André

Flint-Garcia, Sherry

Florez-Rueda, Ana Marcela

Floriani, Talissa d.

Flutre, Timothée

Fogel, Arielle S.

Fondi, Marco

Forest, Thomas

Forsythe, Adrian

Fortwendel, Jarrod

Foster, Toshi M.

Fouquier, Jennifer

Fowler, Emily

Fowler, John E.

Fox, Charles W.

Fox, Donald T.

Francin-Allami, Mathilde

Franco Ortega, Sara

Frandsen, Paul B.

Frank, Margaret

Frantz, Laurent

Freeman, Jules

Frei, Oleksandr

Freitag, Michael

Freitas, Flávia C.

Fricker, Lloyd

Fridman, Eyal

Fritsche-Neto, Roberto

Fritz, Megan L.

Frøkjær-Jensen, Christian

Fu, Jinzhong

Fuchs, Jérôme

Fuhrmann, Nico

Fujita, Akikazu

Fumagalli, Luca

Funda, Tomas

Fundova, Irena

Furrow, Eva

Futcher, Bruce

Gabay-Laughnan, Susan

Gable, Simone

Gable, Simone M.

Gagalova, Kristina K.

Gailing, Oliver

Galagali, Himani Anand

Gallagher, Joseph P.

Gallant, Peter

Gallegos, Maria

Galtier, Nicolas

Galvez, Hayde F.

Ganem, Nabeel

Gao, Ye

Gao, Yuxia

García-Cáceres, Cristina

Garcia-Luis, Jonay

García-Souto, Daniel

Gardiner, Donald

Gartner, Anton

Gasser, Robin

Gassmann, Walter

Gauthier, Jérémy

Gautier, Mathieu

Gaya, Ester

Gaynor, Chris R.

Ge, Wanzhong

Ge, Weihao

Gebelein, Brian

Geck, Renee C.

Geeleher, Paul

Geibel, Johannes

Geiler-Samerotte, Kerry

Genetti, Max

Genuário, Diego

Gepts, Paul

Gerbi, Susan A.

Gerke, Justin P.

Gerstein, Aleeza C.

Gerton, Jennifer L.

Gervais, Nicolas C.

Gezan, Salvador A.

Ghalambor, Cameron

Ghosh, Neha

Ghosh-Roy, Anindya

Giani, Alessandra

Gibson, Amanda K.

Gifford, Danna R.

Gilbert, Clément

Gilbert, Jack

Gilbert, Luke

Gilchrist, Michael A.

Gillespie, Zoe E.

Giordani, Willian

Gioti, Anastasia

Giraud, Tatiana

Gladieux, Pierre

Glauser, Dominique A.

Gleichman, Amy

Glémin, Sylvain

Gluck-Thaler, Emile

Godfrey, R. Keating

Godoy, Camilia

Goh, Hoe-Han

Gohil, Vishal M.

Goig, Galo

Goldberg, Amy

Goldman-Huertas, Benjamin

Goley, Erin D.

Gomes-dos-Santos, André

Gómez Luciano, Luis B.

Gómez-Zapata, Paula

Gompert, Zachariah

GonÃ§alves, Leandro

Goncalves, Carla

Goncalves, Paula

Gondro, Cedric

Gonen, Serap

González-Diéguez, David O.

Goodfellow, Claire

Goodrich-Blair, Heidi

Gordon, Kacy L.

Gorjanc, Gregor

Gorodkin, Jan

Goss, Erica

Gottschalk, Christopher C.

Gottschling, Daniel E.

Götz, Magdalena

Gout, Jean-Francois

Grabowski, Paul P.

Graham, Andrea

Grant, Taran

Gratz, Scott J.

Graves, Jennifer M.

Greenstein, David

Grenier, Cécile

Gresham, David

Griffin, Erik E.

Griffith, Leslie C.

Grigoriev, Igor V.

Gross, Einav

Grover, Corrinne E.

Grueber, Wes

Grueneberg, Alexander

Gruissem, Wilhelm

Gruninger, Robert

Grünwald, Niklaus J.

Gryganskyi, Andrii

Guan, Yongtao

Guerreiro, Marco A.

Guerreiro, Ricardo

Gugger, Muriel

Guiguen, Yann

Guldbrandtsen, Bernt

Gulia-Nuss, Monika

Gunde-Cimerman, Nina

Guo, Lizhong

Guo, Tingting

Guo, Weier

Guo, Yuanwen

Guschanski, Katerina

Gustafsson, Susanne

Gutenkunst, Ryan

Guthrie, Katy

Gutierrez, Lucía

Gutiérrez, Lucía

Haag, Christoph R.

Haas, Fabian B.

Habig, Michael

Hadziabdic, Denita

Hahn, Dan

Haig, David

Haji, Diler

Hajnal, Alex

Halary, Sébastien

Haldar, Devyani

Hale, Joseph J.

Halpin-McCormick, Anna

Hamada, Mayuko

Hamilton, John P.

Hamilton, Matthew G.

Hammerschmidt, Matthias

Han, Li-Hong

Handler, Alfred M.

Hane, James K.

Hanover, John

Hanrahan, Benjamin J.

Hansen, Ole K.

Hao, Haibo

Hao, Nan

Harbison, Susan

Hardy, Nate

Hargreaves, Diana

Hargrove, Amanda

Harkess, Alex

Harpur, Brock A.

Harringmeyer, Olivia

Harrison, Mark

Harrison, Melissa M.

Hart, Anne C.

Hart, Traver

Harting, Rebekkaa

Hashiguchi, Yasuyuki

Haudry, Annabelle

Havill, Nathan P.

Hawlitschek, Oliver

Hawthorne, David

He, Bin Z.

He, Guanghua

He, Zihuai

Hearn, Jack

Hebbar, Prajna

Heckenhauer, Jacqueline

Hedlund, Eva

Heinze, Berthold

Helanterä, Heikki

Helbing, Caren

Henderson, Ashley N.

Henk, Daniel

Hernandez, Ryan D.

Hernando-amado, Sarah

Herrera-Estrella, Alfredo

Herriage, Hunter C.

Herzig, A F.

Hess, Matthias

Hey, Jody

Heyduk, Karolina

Hickey, John M.

Hickey, Lee

Hidalgo, Oriane

Hiller, Michael

Hiltunen Thorén, Markus

Hime, Gary

Hines, Heather M.

Hjelmen, Carl E.

Hoban, Sean

Hobert, Oliver

Hoch, Nicolas

Hochwagen, Andreas

Hoffman, Ellen

Hoffmann, Ary A.

Holland, James B.

Hollis, Brian

Holzer, Astrid S.

Hopper, Keith R.

Hoppler, Stefan

Horianopoulos, Linda

Horn, Rebekah

Horvath, Julie

Hosaka, Kazuyoshi

Hoskins, Aaron

Hou, Jing

Hou, Steven X.

Hou, Tian

Howard, Réka

Howe, Kerstin

Hu, Pei S.

Hu, Yanhui

Hu, Yiming

Hua-Van, Aurelie

Huang, Jun

Huang, Kang

Huang, KC

Huang, Wen

Huang, Yongping

Huang, Yuheng

Huber, Christian D.

Huberman, Joel

Huberman, Lori

Hudson, Matthew

Huerta-Sánchez, Emilia

Hufford, Matthew B.

Hufnagel, Bárbara

Hull, Christina

Hulse-Kemp, Amanda M.

Hundley, Heather A.

Hunnicutt, Kelsie E.

Hunter, Craig P.

Hutton, Samuel F.

Huynh, Bao Lam

Ibrahim, Amir M.

Idnurm, Alexander

Ignatz, Eric

Iino, Yuichi

Ikeda, Akihiro

Ikui, Amy E.

Im, Hae Kyung

Immonen, Elina

Incarnato, Danny

Ingala, Melissa

Ingolia, Nicholas

Ingram, Thomas

Ioannidis, Panagiotis

Iorizzo, Massimo

Irimia, Manuel

Ishaq, Sue

Isik, Fikret

Isobe, Sachiko N.

Iwanicki, Natasha S.

Iwasa, Yoh

Iwata, Hiroyoshi

Iyer, Shruti V.

Jackson, Walker

Jaiswal, Pankaj

James, Timothy Y.

Janbon, Guilhem

Jannink, Jean-Luc

Janss, Luc

Jaramillo-Lambert, Aimee

Jaron, Kamil

Jarquin, Diego

Jarvis, David E.

Jeffares, Daniel C.

Jeffries, Daniel

Jensen, Just

Jeong, Soon-Chun

Jerry, Dean

Jesuthasan, Suresh

Jhuang, Han-Ying

Jiang, Fan

JIang, Yu

Jiao, Renjie

Jiggins, Chris

Jiggins, Francis M.

John, Sona

Johnsen, Pal J.

Johnson, Jeremy

Johnston, J. Spencer

Johnston, Susan E.

Johri, Parul

Jones, Christopher S.

Jones, Corbin

Jordan, David R.

Jose, Antony M.

Juliana, Philomin

Jung, Michaela

Juríková, Katarína

Kadri, Naveen K.

Kaduskar, Bhagyashree D.

Kahman, Regine

Kamfwa, Kelvin

Kämper, Jörg

Kampmann, Martin

Kamsteeg, Erik-Jan

Kannan, Natarajan

Kantanen, Juha

Kantar, Michael B.

Kao, Chen-Hung

Kaplan, Craig D.

Kaplan, Joshua M.

Kapoor, Shilpa

Karaman, Emre

Karasov, Talia

Karin, Ben

Karlsson, Elinor

Karlsson, Magnus

Kasuga, Takao

Katuwal, Mandira

Katz, Martin L.

Kaur, Maninder

Kause, Antti

Kayal, Ehsan

Kayser, Matthew

Ke, Yi-Hong

Keeling, Christopher I.

Kelleher, Jerome

Kelley, Scott

Kellogg, Elizabeth A.

Kelly, Libusha

Kelsey, Gavin D.

Kennedy, Scott

Kennell, Jennifer

Kent, Tyler

Kenworthy, Kevin

Kern, Andrew D.

Kerney, Ryan

Kesiime, Eunice

Khakh, Baljit

Khalturin, Konstantin

Kharrat, Magda

Kianian, Shahryar F.

Kick, Daniel R.

Kim, Hongkyun

Kim, John K.

Kim, Ki-Tae

Kim, Kyuhyung

Kim, Taehyeung

Kimelman, David

Kimmel, Charles

King, Elizabeth G.

Kippes, Nestor

Kirisits, Thomas

Kistler, Logan

Kitchen, Sheila A.

Kitsou, Chrysoula

Kizil, Caghan

Kjærner-Semb, Erik

Klápště, Jaroslav

Klemm, Jacob

Klocko, Andrew D.

Klure, Dylan

Kobayashi, Akira

Kobayashi, Takehiko

Koch, Jonathan B.

Koch, Marcus A.

Koch, Robert L.

Kochian, Leon V.

Kocot, Kevin M.

Kodama, Miyako

Koepfli, Klaus-Peter

Koloski, Cody

Kondo Rodriguez, Demian Takumasa

Konkel, Miriam

Konstantinides, Nikos

Koop, Ben F.

Kopp, Artyom

Kopp, Jeffrey

Korb, Judith

Korneliussen, Thorfinn S.

Kornfeld, Kerry

Koromila, Theodora

Kortel, Nadine

Kortsinoglou, Alexandra M.

Kosch, Tiffany

Koski, Matthew

Kothe, Erika

Kotze, Andrew

Kouvelis, Vassili N.

Krabbenhoft, Trevor J.

Kraemer, Brian

Kraft, Florian

Krantz, David E.

Krapf, Patrick

Krasileva, Ksenia V.

Krasovec, Marc

Kratochvil, Lukas

Krätschmer, Ilse

Krause, Margaret R.

Krause, Michael W.

Kreth, Jens

Krogan, Nevan

Kronmiller, Brent

Kropp, Peter

Kruth, Perryn S.

Kruuk, Loeske E.

Kuhl, Susanne

Külheim, Carsten

Kurant, Estee

Kuraparthy, Vasu

Kurta, Khrystyna

Kurtz, Joachin

Kusmec, Aaron

Kuzmin, Elena

Kwan, Elizabeth X.

Kyriazis, Chris

L'Etoile, Noelle D.

LÃ³pez-Urrutia, Eduardo

LaBonne, Carole

Lachance, Joseph

Lacin, Haluk

Lado, Bettina

Lamichhaney, Sangeet

Lamont, Ian

Landry, Christian R.

Lanfear, Rob

Lange, Bernd M.

Langer, Gerald

Large, Christopher R.

Larracuente, Amanda M.

Larschan, Erica N.

Larsen, Peter

Larson, Steve R.

Larti, Farzaneh

Larzul, Catherine

Lasky, Jesse R.

Lassmann, Hans

Laurent, Stefan

Lavrov, Dennis

Lawson, Heather A.

Lazaridis, Iosif

Lazzaro, Brian P.

Leaché, Adam D.

Leakey, Andrew D.

Leal-Dutra, Caio A.

Lecomte, Laurie

LeDeaux, John

Lee, Dung-Fang

Lee, Hanbin

Lee, Junho

Lee, Siu S.

Lee, Sonny T.

Leeb, Tosso

Leebens-Mack, James

Leese, Florian

Leeuw, Christiaan d.

Legarra, Andres

Legouis, Renaud

Legras, Jean-Luc

Legried, Brandon J.

Lehmann, Philipp

Lehmann, Robert

Lehoczky, Jessica

Lei, Chuzhao

Lei, Lihong

Leiser, Willmar L.

Lemos, Bernardo

Lenstra, Johannes A.

Leroy, Thibault

Leung, Yuk F.

Levesque, Roger

Levi, Amnon

Levings, Megan

Lewellyn, Lindsay

Lewin, Gary

Lewis, Katharine E.

Lewis, Morag A.

Lewis, Zachary A.

Li, Chenhong

Li, Fei

Li, Jia-Tang

Li, Kelly Y.

Li, Sheng

Li, Xin

Li, Yuemin

Li, Ze

Li, Zheng

Liang, Zhikai

Liao, Bo-Kai

Liber, Julian

Liebau, Eva

Lillemo, Morten

Lim, Daniel

Lin, Kejian

Lin, Zhongxu

Lindblad-Toh, Kerstin

Lindsey, Amelia R.

Ling, Jiaqiang

Linnen, Catherine R.

Linscott, T. Mason

Lipka, Alexander E.

Lisby, Michael

Lisch, Damon

Liu, Gao-Qiang

Liu, Huan

Liu, Jianquan

Liu, Jingyu

Liu, Jingze

Liu, Qikun

Liu, Shikai

Liu, Xianan

Liu, Yilun

Liu, Zhipeng

Liu, Zhong-Jian

Liu, Zukai

Ljungdahl, Per

Ljungdahl, Per O.

Llaca, Victor

Llorente, Bertrand

Lloret Fernández, Carla

Lo, Nathan

Loarca, Jenyne

Lobet, Guillaume

Locke, John

Lockridge Mueller, Rachel

Loftus, Mark

Logan, Mary

Long, Anthony D.

Long, Erping

Long, Evan M.

Lopes Filho, Erick A.

Loppin, Benjamin

Lorenz, Aaron

Lorrain, Cecile

Lourenco, Daniela

Lowery, Laura

Lozier, Jeffrey D.

Lu, Dabao S.

Lukubye, Ben

Lumbsch, Thorsten

Lund, Mogens S.

Lundquist, Erik A.

Luo, Jian

Luo, Xia

Luo, Yan

Luo, Yi-Jyun

Luo, Yichen

Lürig, Moritz

Luschnig, Stefan

Lux, LeAnn

Lux, Thomas

Lyons, Jessica B.

Lyons, Leslie A.

Lysøe, Erik

Ma, Li-Jun

Macabenta, Frank

Macdonald, Stuart J.

Mace, Emma

Machado, Carlos A.

MacKay, John J.

Mackintosh, Carl J.

MacLean, Adam L.

MacNeil, Michael D.

Macqueen, Daniel J.

Madden, Lawrence

Madhani, Hiten D.

Madhu, Bhoomi

Maduro, Morris F.

Magadi, Srivathsa S.

Magalhaes, Jurandir V.

Magbanua, Zenaida V.

Maggert, Keith A.

Magwene, Paul M.

Mahmoud, Mohamed A.

Maia, Luciano C.

Majumder, Rajib

Makeyev, Eugene

Malik, Harmit S.

Malinda, Raj

Mallard, François

Maloof, Julin N.

Malukiewicz, Joanna

Mancera, Eugenio

Mancuso, Nicholas

Mandák, Bohumil

Mandeville, Elizabeth G.

Manglai, Dugarjaviin

Mango, Susan

Mangone, Marco

Maniego, Jillian

Manna, Debraj

Manousaki, Tereza

Mao, Shaoli

Marais, Gabriel A.

Marand, Alexandre P.

Marie, Benjamin

Maroilley, Tatiana

Marshall, John

Marsit, Souhir

Martin, Francis M.

Martínez, Paulino

Martí­nez-Alesón Garcia, Paloma

Martins, Tiago

Martinson, Ellen O.

Masel, Joanna

Masly, John P.

Mason, Charles J.

Masonbrink, Rick

Massel, Karen

Mathers, Thomas C.

Mathies, Laura D.

Mathieson, Iain

Mathieu, Laura

Matreyek, Kenneth A.

Matthews, Ben

Maughan, Peter J.

Mauleon, Ramil

Mauthner, Stephanie

Mazo-Vargas, Anyi

McBride, Carolyn S.

McCarthy, Charley G.

McClure, Allison

McDonald, Jeanne M.

McElroy, Kyle E.

McEvoy, Susan L.

McGaugh, Suzanne

McGhee, James D.

McGrath, J M.

McGrath, Patrick T.

McIntyre, Lauren M.

McJunkin, Katherine

McKay, Daniel J.

McKay, John

McKenna, Duane D.

McKim, Kim S.

McPherson, Jeanne-Marie E.

McVean, Gilean

Medeiros, Daniel

Medina, Monica

Medugorac, Ivica

Megraw, Timothy

Meisel, Richard P.

Mello, Claudio

Mello, Craig C.

Melzer, Rainer

Memisoglu, Gonen

Mendes, Fabio

Mendoza, Hector

Meng, Junlong

Mera, Paola E.

Merritt, Justin

Merritt, Thomas J.

Metcalf, Jessica L.

Meyer, Damon

Mhora, Terence T.

Michael, Todd P.

Michel, Andrew

Miga, Karen

Miga, Karen H.

Miglior, Filippo

Milán, Marco

Miles, Timothy

Miller, Danny E.

Miller, Matthew J.

Min, Jaewon

Miron, Waldir

Missaoui, Ali

Mitchell, Aaron P.

Mitchell, Jennyfer M.

Mitina, Aleksandra

Mitter, Neena

Miura, Osamu

Miyadera, Keiko

Mizumoto, Kota

Moen, Thomas

Moens, Cecilia B.

Moest, Markus

Moffat, Jason

Mohr, Stephanie E.

Molina Pineda, Julio A.

Mollinari, Marcelo

Momen, Mehdi

Montell, Craig

Montes, Chris

Montesinos-López, Osval A.

Monteverde Dominguez, Eliana

Montgomery, Tai

Montooth, Kristi L.

Moolhuijzen, Paula

Mooney, Jazlyn A.

Mora, Pablo

Moragues, Maria-Dolores

Morales-Cruz, Abraham

Moreau, Laurence

Morel, Jean-Benoit

Morgan, Aaron

Morgante, Fabio

Morota, Gota

Morran, Levi T.

Morrell, Peter

Morrison, Maike L.

Morton, Elizabeth A.

Mosca, Timothy J.

Moscou, Matthew

Mosimann, Christian

Mottola, Austin

Moyers, Brook T.

Muhr, Jonas

Mukherjee, Shreyasi

Müller, Jürg

Mumm, Rita H.

Munoz, Patricio R.

Muñoz-Amatriaín, Maria

Munyard, Kylie

Mural, Ravi V.

Muralidhar, Pavitra

Murani, Eduard

Murga-Moreno, Jesús

Murphy, Kevin

Murphy, Matthew D.

Murphy, William

Murray, Steve

Mussmann, Steven M.

Mutalik, Vivek

Muyle, Aline

Myers, Chad L.

Myers, Edward A.

Nadeau, Simon

Nagano, Atsushi J.

Nageshan, Rishi K.

Nagy, Laszlo

Naidoo, Vinny

Naish, Kerry A.

Naktang, Chaiwat

Nallari, Pratibha

Narbona, Eduardo

Nath, Avindra

Navarro, Rosa E.

Nave, Klaus-Armin

Nehring, Volker

Nelms, Brad

Nelson, Matthew D.

Nesculea, Anamaria

Neuenkirchen, Nils

Newcomb, Susan

Neyhart, Jeffrey

Ng'oma, Enoch

Nguyen Ba, Alex N.

Ni, Jing

Nicke, Annette

Nickoloff, Jac A.

Nielsen, Matthew

Nielsen, Rasmus

Nielson, Kirsten

Nik-Zainal, Serena

Nilsson, Jakob

Nisaa, Khairun

Nitschko, Volker

Nock, Catherine J.

Noel, EMILY

Noguchi, Eishi

Nolan, Tony

Nonet, Michael L.

Normark, Benjamin B.

Norris, Adam

Ntoukakis, Vardis

Nuckolls, Nicole

Nyholt, Dale R.

O'Connor-Giles, Kate M.

O'Donoghue, Patrick

O'Keeffe, Catherine

O'Leary, Collin A.

Oboh, Mary A.

Ohlsson, Åsa

Ohm, Robin A.

Ojeda, Jonathan

Ojha, Arjun

Olsen, Kenneth M.

Olson, Åke

Olsson, Sanna

Olzmann, James

Oostra, Vicencio

Opulente, Dana A.

Orlando, Ludovic

Otaegui, David

Ottesen, Elizabeth A.

Ottoni, Claudio

Ou, Guangshuo

Overy, David P.

Paddock, Kyle

Paffetti, Donatella

Pal, Utpal

Palmer, Daniela

Palti, Yniv

Palu, Rebecca A.

Pan, Chun-Liang

Pandey, Gunjan

Pannebakker, Bart A.

Papa, Roberto

Papa, Yvan

Papathanos, Philippos

Pardo-Manuel de Villena, Fernando

Parent, Christine

Park, Yong-Jin

Parkinson, Christopher

Parkinson, John

Parrish, Jay

Parvez, Suhel

Pasman, Joelle

Patel, Hardip R.

Patel, Jinesh

Patel, Maulik R.

Patel, Roshni A.

Paterson, Adrian

Patil, Gunvant B.

Pauli, Duke

Paulose, Jayson

Paulson, Amber R.

Payne, Joshua L.

Pearce, Stephen

Pearson, John

Peck, Lily

Pedersen, Mikkel Winther

Pees, Christiane

Pellegrino, Mark

Pemberton, Josephine M.

Peng, Ting

Pereira, Andy

Pereira, Guilherme d.

Perez, Bruno C.

Perez, Olga

Pérez-Rodríguez, Paulino

Perner, Jan

Perron, Muriel

Perry, Keith

Peter, Benjamin M.

Petersen, Jessica

Petersen-Jones, Simon

Petrullo, Lauren

Pezzolesi, Marcus

Pfeifer, Susanne P.

Pfennig, Aaron

Pfenninger, Markus

Phifer-Rixey, Megan

Phillips, Sarah R.

Piepho, Hans-Peter

Pierce, Jonathan T.

Pinto, Brendan J.

Pirani, Renata M.

Pires, J. Chris

Pirovani, Carlos Priminho

Platre, Matthieu P.

Pleasure, Samuel

Pletzer, Dan

Plomion, Christophe

Pocock, Roger

Pocrnic, Ivan

Poets, Ana M.

Pöggeler, Stefanie

Polashock, James

Ponomarova, Olga

Ponsuksili, Siriluck

Pool, John E.

Pope, Nathaniel

Popescu, Flaviu

Port, Fillip

Portik, Dan

Posthuma, Danielle

Postlethwait, John H.

Postma, Joeke

Potter, Adam

Powell, Daniel

Preger-Ben Noon, Ella

Priya, Rashmi

Program, Peer R.

Pronk, Jack

Pruefer, Kay

Przanowska, Roza K.

Przeworski, Molly

Pucker, Boas

Puckett, Emily

Pujol, Marta

Pujol, Nathalie

Puopolo, Gerardo

Puri, Dharmendra

Puritz, Jonathan

Pyron, Alex

Qiu, Yinjie

Queitsch, Christine

Quignon, Pascale

Quillet, Edwige

Quintana, Francisco Javier

Raab, Jesse R.

Raffo, Miguel Angel

Rafter, Pierce

Rahit, K. M. Tahsin Hassan

Rainey, Katy M.

Rajeev, Sanjana

Ramachandran, Sohini

Ramadoss, Niveditha

Ramakers, Jip J.

Ramamurthi, Kumaron

Ramos-Madrigal, Jazmin

Ramsey, Jolene

Ramstein, Guillaume

Rankin, Catharine H.

Rasgon, Jason L.

Raudsepp, Terje

Ravi, Samathmika

Ray, David

Ray, Rishav

Rayamajhi, Niraj

Reams, Andrew B.

Rebetzke, Gregory

Reed, Laura K.

Regev, Aviv

Reis, Tânia

Reis-Cunha, João Luís

Reitzel, Adam M.

Remsburg, Carolyn

Rentzsch, Philipp

Reuter, Max

Reverter, Antonio

Rhen, Turk

Ricard, Anne

Rice, Brian

Rice, Gavin

Richards, Stephen

Richardson, William

Richmond, Janet E.

Riddle, Nicole C.

Rideout, Elizabeth J.

Riera, Celine

Rincent, Renaud

Rio, Simon

Rios, Esteban

Rissi, Daniel V.

Ritchie, Michael G.

Rivera-Colón, Angel G.

Robbins, Kelly R.

Robbins, Matthew D.

Robert, Claude

Roberts, Philip A.

Robinson, Aaron J.

Rocheleau, Christian E.

Rochus, Christina

Rockman, Howard A.

Rockman, Matthew V.

Rodrigues, Murillo F.

Rodriguez, Moira

Rodriguez de la Vega, Ricardo

Rodriguez Morales, Roberto E.

Rodríguez-Beltrán, Jerónimo

Roe, Michael

Rog, Ofer

Rogers, Aric N.

Rogers, David

Rogers, Rebekah L.

Rohde, Palle Duun

Rohrlach, Adam B.

Roldán-Ruiz, Isabel

Rolland, Stéphane G.

Rollinson, David

Rollmann, Stephanie M.

Ronnegard, Lars

Rose, Lindy J.

Rosenberg, Noah A.

Rosendale, Andrew

Ross, Colin J.

Ross-Ibarra, Jeffrey

Rossini, Laura

Roth, Cullen

Roth, Frederick P.

Roth, Lukas

Rothi, Hafiz

Rothman, Joel

Rougemont, Quentin

Rouskin, Silvi

Roy, Richard

Ru, Susan

Ruan, Donglin

Ruane, Sara

Ruden, Douglas M.

Ruff, Emily

Ruiz-Ruano, Francisco J

Rungis, Dainis

Rustgi, Sachin

Ruta, Vanessa

Rutkoski, Jessica

Rutkoski, Jessica E.

Ruud, Anja K.

Ruvinsky, Ilya

Ryan, Joseph

Ryu, Taewoo

Saayman, Xanita

Saber, Sayran

Sachinidis, Agapios

Sachs, Matthew S.

Saenko, Suzanne V.

Sagasti, Alvaro

Saher, Gesine

Sakurai, Kengo

Salas-Fernandez, Maria G.

Sallam, Ahmad

Salson, Marine

Salzberg, Steven L.

Sampaio, Ana

Samuk, K.

Sanabria, Ryo

Sánchez, Juan A.

Sánchez, Leopoldo

Sanchez-Munoz, Raul

Sanchez-Roige, Sandra

Sanetomo, Rena

Sanjana, Neville

Santalla, Marta

Santantonio, Nicholas

Santos-Lopez, Alfonso

Sanyal, Suparna

Sapir, Yuval

Sapkota, Surya

Sargent, Daniel J.

Sasaki, Eriko

Satake, Akiko

Sato, Ken

Satoh, Akira

Sattler, Scott

Savo Sardaro, Maria Luisa

Sawers, Ruairidh J.

Saxena, Ayush S.

Scally, Aylwyn

Schaack, Sarah

Schacherer, Joseph

Schachtschneider, Kyle

Schal, Coby

Scheben, Armin

Schedl, Tim

Scheffler, Brian E.

Schenkel, Flavio S.

Schepler-Luu, Van

Schetelig, Marc F.

Schiabor Barrett, Kelly M.

Schiffels, Stephan

Schilbert, Hanna

Schlupp, Ingo

Schlüter, Andreas

Schmidt, Anja

Schmidt, Tom

Schmitt, Thomas

Schmitz, Robert J.

Schmutz, Jeremy

Schmutz, Jeremy J.

Schnable, James C.

Schneeberger, Korbinian

Schneider, David A.

Schnitzler, Christine

Schnorrer, Frank

Schoborg, Todd

Schoelz, James

Schön, Chris-Carolin

Schook, Larry

Schoville, Sean

Schraiber, Joshua G.

Schrider, Daniel R.

Schulenburg, Hinrich

Schulte-Hostedde, Albrecht

Schultz, Darrin T.

Schulz, Aimee J.

Schumacher, Julia

Schwabe, Enrico

Schwartz, John

Schweizer, Rena M.

Schwessinger, Benjamin

Scofield, Douglas G.

Scott, Ian

Scott, Jacob G.

Scott, Maxwell J.

Sedano-Cruz, Raul

Seemann, Stefan

Seemann, Torsten

Segura, Vincent

Sehr, Eva M.

Seidel, Hannah S.

Seidl, Michael F.

Sekelsky, Jeff

Serrano, Antonio

Servedio, Maria

Servin, Bertrand

Sethuraman, Arun

Seydoux, Geraldine

Shaffer, Sydney

Shakes, Diane C.

Shankar, Vijay

Shapiro, Beth

Shapiro, Rebecca S.

Sharbel, Timothy

Sharbrough, Joel

Sharma, Nimisha

Sharp, Andrew

Sharp, Nathaniel P.

Sheat, Samar

Shefali, Shefali

Sheltzer, Jason

Shen, Kang

Shendure, Jay

Sherlock, Gavin

Sherwood, David

Shi, Mengwei

Shik, Jonathan

Shirasawa, Kenta

Shrestha, Padmina

Shultz, Allison J.

Si, Yaqin

Si, Yichen

Sideli, Gina

Sigwart, Julia

Silke, John

Sillanpää, Mikko J.

Sillo, Fabiano

Silva Pereira, Cristina

Silver, Luke W.

Sim, Sheina B.

Simakov, Oleg

Simard, Martin J.

Simon, Alexis

Simpson, Elizabeth M.

Singh, Nadia D.

Singh, Varsha

Singson, Andrew

Skeath, James B.

Skeffington, Alastair

Skrubbeltrang Hansen, Laura

Slate, Jon

Sless, Trevor J.

Sloan, Daniel B.

Slotte, Tanja

Smanski, Michael J.

Smart, Brian C.

Smith, Damon

Smith, Megan L.

Smith, Parker

Smith, Shane

Smith, Shavannor M.

Smith-Bolton, Rachel K.

Smolikove, Sarit

Snelders, Eveline

Sohail, Mashaal

Sohn, Soo-In

Sonam, Surabhi

Song, Hojun

Song, Zhaobin

Sork, Victoria L.

Sorrells, Mark E.

Spanner, Rebecca

Sparks, Michael E.

Spatafora, Joseph W.

Spence, Jeffrey P.

Sperling, Felix A.

Spigler, Rachel

Srinivasan, Jagan

Sserumaga, Julius P.

Stahl, Eli A.

Stahlke, Amanda R.

Stajich, Jason E.

Staller, Max V.

Stam, Remco

Stankowski, Sean

Staton, Margaret

Stearns, Frank

Steenackers, Hans

Steffenson, Brian J.

Steiner, Cynthia

Steiner, Florian A.

Steinhauer, Josefa

Steinrücken, Matthias

Stephens, Timothy

Stern, Michael

Sternberg, Paul W.

Stevens, Lewis

Stewart, Jane E.

Stift, Marc

Stolle, Eckart

Storchova, Helen

Stranden, Ismo

Stuart, David T.

Stuart, Rosemary

Stuckert, Adam M.

Su, Zhengchang

Suffert, Frederic

Sugimoto, Katsunori

Suh, Alexander

Sun, Cheng

Sun, Sheng

Sundaram, Meera V.

Suvorov, Anton

Swarts, Kelly

Sweeney, Patrick

Swingle, Bryan

Switzer, Alexandra

Syeda, Aisha

Symula, Rebecca

Szymański, Jędrzej

Tabima, Javier F.

Tagirdzhanova, Gulnara

Tai, Helen

Talbot, Samuel C.

Tallada, Víctor A.

Tanguy, Arnaud

Taniguti, Cristiane H.

Tarailo-Graovac, Maja

Tarone, Aaron M.

Taubert, Stefan

Taylor, Scott

Taylor, Susan S.

Taylor, Véronique

Taylor-Wardell, Samuel

Technau, Ulrich

Teets, Nicholas

Teh, Soon

Temple, Seth

Tennessen, Jason M.

Terrier, Nancy

Teshome, Abel

Teske, Peter

Tessele, Augusto

Teterina, Anastasia A.

Teves, Sheila S.

Texada, Michael J.

Theis, Martin

Thomas, Ariane E.

Thomas, Laura

Thomas Tate, Ann

Thompson, Addie M.

Thompson, Andrew W.

Thornton, Kevin R.

Threadgill, David W.

Thumma, Bala R.

Tian, Ai-Guo

Tian, Miaoying

Tibbits, Josquin

Timmermans, Martijn

Timp, Winston

Ting, Nelson

Tiso, Till

Tissenbaum, Heidi

Tittes, Silas

Tiwari, Vijay

Tobias, Peri A.

Todd, Richard B.

Toghiani, Sajjad

Tolhurst, Daniel

Tollis, Marc

Tolman, Ethan R.

Tonnabel, Jeanne

Tontonoz, Peter

Toomajian, Christopher

Tootle, Tina L.

Topp, Christopher N.

Torres-Sánchez, Maria

Toth, Amy

Toups, Melissa A.

Tourrette, Elise

Toussaint, Emmanuel F.

Toyoda, Atsushi

Tracey, W. Daniel

Trainor, Paul

Traut, Walther

Trevelline, Brian

Triant, Deborah

Troggio, Michela

Tsai, Chung-Jui

Tsairidou, Smaragda

Tsang, Adrian

Tsigenopoulos, Costas S.

Tuck, Simon

Tuinstra, Mitch

Tulpule, Asmin

Turelli, Michael

Turner-Hissong, Sarah D.

Tzelepis, Georgios

Tzika, Athanasia C.

U'Ren, Jana

Uchiyama, Hideho

Udall, Joshua A.

Uebel, Celja J.

Uh, Hae-Won

Ünal, Elçin

Unckless, Robert L.

Updike, Dustin L.

Usadel, Bjoern

Usadel, Björn

Uy, J. Albert

Vallebueno-Estrada, Miguel

Vallejo, Roger L.

Valls, Marc

Van Buskirk, Cheryl

Van Dam, Matthew

Van Delden, Christian

Van den Heuvel, Joost

Van Eeuwijk, Fred A.

Van Munster, Jolanda

Van Steenbeek, Frank

VanBuren, Robert

Vandebergh, Marijne

Vanderzalm, Pam

Varaldi, Julien

Vargas-Rodriguez, Oscar

Vasconcelos, Maria L.

Vasemägi, Anti

Vaughan, Sue

Vaughn, Andrew H.

Velasco, Vera M.

Velazco, Julio

Veller, Carl

Veltsos, Paris

Vendramin, Giovanni G.

Venkataraman, Pavithra

Vereecken, Nicolas

Verhoeven, Koen

Via, Sara

Vicoso, Beatriz

Vidal-Dupiol, Jeremie

Vieira, Maria L.

Vierbuchen, Thomas

Vigouroux, Yves

Vila, Roger

Villa, Roger

Villarreal Aguilar, Juan Carlos

Villwock, Seren S.

Vining, Kelly J.

Vischioni, Chiara

Vitale, Paolo

Vizueta, Joel

Vogan, Aaron A.

Vogel, Kevin J.

Vogl, Claus

Vokes, Steven

Voolstra, Christian R.

Voronina, Ekaterina

Voss-Fels, Kai P.

Voytas, Dan

Wachowiak, Witold

Wacker, Theresa

Waddell, Nicola

Wade, Claire M.

Wadsworth, Crista

Wagner, Andreas

Wagner, Eric

Wahls, Wayne P.

Wainberg, Michael

Waku, Tsuyoshi

Waldman, Alan S.

Waldmann, Patrik

Walhout, Marian

Walker, Amy K.

Walkley, Carl

Wall, Jeffrey D.

Wallace, Jason G.

Wanelik, Klara

Wang, Di

Wang, Hantao

Wang, Huaisong

Wang, Jade

Wang, Jen-Yu

Wang, Jiongming

Wang, Jiou

Wang, Lisa

Wang, Qian

Wang, Wei

Wang, Wen-Ming

Wang, Yan

Wang, Yiguan

Wang, Yuanyuan

Wang, Yuxuan

Wang, Zhao-Wen

Wang, Ziyan

Ward, Jordan D.

Ward, Robert E.

Ware, Doreen

Warthmann, Norman

Washburn, Jacob D.

Watanabe, Kyoko

Watanabe, Yoshinori

Waterhouse, Robert M.

Watkins, Eric

Watsa, Mrinalini

Watts, Jennifer L.

Wedger, Marshall

Weeks, Kevin M.

Wei, Kevin

Wei, Wei

Wei, Xing H.

Wei, Xinzhu

Wei, Yuming

Wei, Yun

Weinreich, Daniel

Weiss, Brian L.

Weiss, Eric

Weisweiler, Marius

Wells, Kristen

Weng, Mo

Weng, Yi-Ming

Wenzell, Katherine

Werren, John H.

Westram, Anja Marie

White, Benjamin

White, Rob

Whiteman, Noah K.

Whiting, Michael

Wiberg, R. Axel W.

Wicky, Chantal

Wilberg, Ragnhild

Wildonger, Jill

Wilker, Jennifer

Willsey, Helen

Wilusz, Jeremy

Wimalanathan, Kokulapalan

Winata, Cecilia

Winchell, Kristin

Windbichler, Nikolai

Wingfield, Brenda D.

Wink, Michael

Wirsel, Stefan

Wisecaver, Jennifer H.

Wisser, Randall J.

Witte, Thomas E.

Wolfe, Kenneth H.

Wolfe, Marnin D.

Wölfl, Benjamin

Wolfner, Mariana F.

Wollenberg Valero, Katharina

Wong, Adam

Wong, Thomas

Woodruff, Gavin C.

Worsaae, Katrine

Worthington, Margaret L.

Wrasman, Kristie

Wright, Charlotte

Wu, Joseph C.

Wu, Yanqi

Wulff, Brande B.

Würschum, Tobias

Wurzinger, Maria

Xavier, Alencar

Xavier, Joao

Xie, Baogui

Xiong, Liang

Xu, Dawei

Xu, Jianping

Xu, Junxiang

Xu, Shaolin

Xu, Shuqing

Xu, X.Z. Shawn

Xu, Yuyan

Yadegari, Ramin

Yan, Hua

Yan, Jianbing

Yan, Lang

Yan, Shuxun

Yan, Ying

Yáñez, José M.

Yang, Kevin

Yang, Rebecca C.

Yang, Sihai

Yang, Xiang-Lei

Yang, Xiaofei

Yang, Xiuqing

Yang, Yongjie

Yanowitz, Judith

Yap-Chiongco, Meghan

Yasuto, Ishii

Ye, Bing

Ye, Guoyou

Yeaman, Sam

Yelina, Nataliya E.

Yokoyama, Hitoshi

You, Ping

Youssef, Noha H.

Yu, Daoyuan

Yu, Fuqiang

Yu, Hao

Yu, Hong

Yu, Jianming

Yuan, Hao

Yuksel, Mete K.

Zahler, Alan M.

Zamberlam, Gustavo

Zappia, Maria Paula

Zappulla, David C.

Zeis, Bettina

Zeitlinger, Julia

Zelcer, Noam

Zeng, Zhao-Bang

Zetka, Monique

Zhang, Baolin

Zhang, Bin

Zhang, Bo

Zhang, Dabing

Zhang, De Y.

Zhang, Deqiang

Zhang, Feng

Zhang, Hechen

Zhang, Hong

Zhang, Huiting

Zhang, Jinfa

Zhang, Jinjing

Zhang, Lingling

Zhang, Ren-Gang

Zhang, Yun

Zhao, Li

Zhao, Tianjing

Zhao, Yunxia

Zhong, Shengqiang

Zhou, Chenxi

Zhou, Qi

Zhou, Ran

Zhou, Rui

Zhou, Xiang

Zhou, Xiaoxue

Zhou, Yongfeng

Zimin, Aleksey V.

Zimmer, Alex

Zinovyeva, Anna Y.

Zorn, Aaron M.

Zuryn, Steve

